# Safety and efficacy of 0.02% and 0.01% atropine on controlling myopia progression: a 2-year clinical trial

**DOI:** 10.1038/s41598-021-01708-2

**Published:** 2021-11-15

**Authors:** Can Cui, Xiujuan Li, Yong Lyu, Li Wei, Bingxin Zhao, Shiao Yu, Junbo Rong, Yanhui Bai, Aicun Fu

**Affiliations:** The First Affiliated Hospital of Zhengzhou University, No. 1 Jianshe Road, Zhengzhou, 450000 China

**Keywords:** Refractive errors, Randomized controlled trials

## Abstract

Four hundred myopic children randomly received atropine 0.02% (n = 138) or 0.01% (n = 142) in both eyes once-nightly or only wore single-vision spectacles (control group) (n = 120) for 2 years. Spherical equivalent refractive error (SER), axial length (AL), pupil diameter (PD), and amplitude of accommodation (AMP) were measured every 4 months. After 2 years, the SER changes were − 0.80 (0.52) D, − 0.93 (0.59) D and − 1.33 (0.72) D and the AL changes were 0.62 (0.29) mm, 0.72 (0.31) mm and 0.88 (0.35) mm in the 0.02% and 0.01% atropine groups and control group, respectively. There were significant differences between changes in SER and AL in the three groups (all *P* < 0.001). The changes in SER and AL in the 2nd year were similar to the changes in the 1st year in the three groups (all *P* > 0.05). From baseline to 2 years, the overall decrease in AMP and increase in PD were not significantly different in the two atropine groups, whereas the AMP and PD in the control group remained stable (all *P* > 0.05). 0.02% atropine had a better effect on myopia control than 0.01% atropine, and its effects on PD and AMP were similar to 0.01% atropine. 0.02% or 0.01% atropine controlled myopia progression and AL elongation synchronously and had similar effects on myopia control each year.

## Introduction

The prevalence of myopia is significantly increasing worldwide, especially in Asia^1–3^. It is predicted that by 2050, about half of the population will be suffering from myopia; as many as 10% of the cases are expected to be of high myopia^4^. High myopia and excessive eye growth can cause sight-threatening ocular complications, resulting in a huge socio-economic burden^5,6^.

Several studies, including those carried out in Singapore, mainland China, Hong Kong China and other countries, have shown that moderate- and low-concentration atropine eye drops (e.g., 0.01%, 0.025%, 0.05%) may effectively and safely slow the progression of myopia in children^7–18^.However, most of these studies, including our previous study^18^, were 1-year short term follow-up studies, except the 5-year follow-up study in Singapore ( Atropine for the Treatment of Myopia, ATOM), 2-year follow-up studies in Hong Kong China (Low-Concentration Atropine for Myopia Progression, LAMP) and in the United States^8,11,12^.

Notably, in the ATOM2 study^7^, 0.01% atropine had comparable efficacy in controlling myopia progression with 0.1% and 0.5% atropine. Moreover, 0.01% atropine was more effective in the second year than in the first year. The changes in axial length (AL) and spherical equivalent refractive error (SER) were not synchronous; the degree of myopia was stable, while AL continued to increase between 8 and 24 months after using 0.01% atropine. However, these phenomena were not observed with higher concentrations of atropine (0.1% or 0.5%). During the second-year observation in the LAMP study^11^, which lacked a control group, low-concentration atropine had a dose-related effect on myopia control. The efficacy of 0.05% atropine was double than that of 0.01% atropine. Compared with the 1st year, the 2nd year efficacies of 0.05% and 0.025% atropine were similar, with a mild improvement in the 0.01% atropine group. Also, 0.01%, 0.025%, and 0.05% atropine controlled both myopia progression and AL elongation. In a 2-year retrospective study in the US^12^, 0.01% atropine had a better effect on myopia control during the second year than during the first year. However, only refractive error, but not AL, was measured in that study.

Our 1-year study found that 0.02% atropine had a better effect on myopia control than 0.01% atropine, but 0.02% and 0.01% atropine showed similar side-effects^18^. This 2-year study was a continuation of our previous 1-year study^18^, which was aimed at answering the following questions: Is the long-term use of low-dose atropine effective, safe, and dose-dependent in controlling myopia progression in children? We also investigated difference in efficacies between 0.02 and 0.01% atropine and whether low-dose atropine controlled myopia progression and AL elongation synchronously in mainland China.

## Results

**Figure 1:**
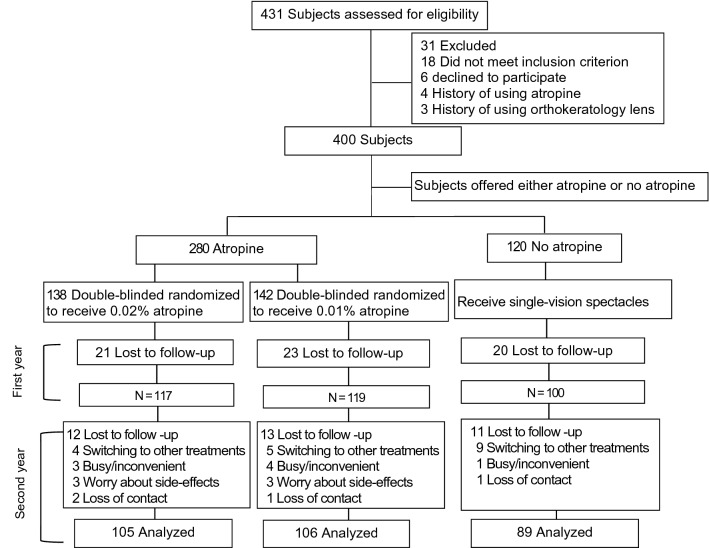
Subject recruitment and randomization flowchart.

Among the 400 children enrolled, 64 were lost to follow-up within the first year, and 336 (84%) continued to participate in the extended trial. There were 117, 119, and 100 children in the 0.02% and 0.01% atropine and control groups, respectively (Fig. 1). At baseline, age, sex, body mass index (BMI), SER, IOP, pupil diameter, AMP, AL, ACD, corneal curvature, time spent in outdoor activity and near work, and parental myopia status were similar among the groups, with no significant differences (Table 1). Children who were lost to follow up or withdrew participation were excluded from analyses. Reasons for withdrawal included being too busy or finding the trial inconvenient, worry about side-effects, enrolling in other trials, and difficulty in applying eye drops. No significant differences were noted between the baseline parameters of the 336 subjects who completed the study and the 64 subjects who dropped out during the first year (*P* > 0.05). Furthermore, the baseline parameters of the 300 subjects who completed the 2 years of follow-up were similar to those of the 100 subjects who did not (Table 1).

**Table 1:** Baseline characteristics of study participants who completed 2 years versus those who have not completed 2 years.

Variables	Completed 2 years (N = 300)				Not completed 2 years (N = 100)			
	0.02% atropine N = 105	0.01% atropine N = 106	Control group N = 89	*P*	0.02% atropine N = 33	0.01% atropine N = 36	Control group N = 31	*P*
	Mean ± SD	Mean ± SD	Mean ± SD		Mean ± SD	Mean ± SD	Mean ± SD	
Age (year)	9.6 ± 1.8	9.4 ± 1.7	9.3 ± 1.4	0.56	9.3 ± 2.1	9.2 ± 2.5	9.6 ± 2.3	0.66
Sex (male, n and %)	55 (52.4%)	55 (51.9%)	47 (52.8%)	0.99	18 (54.5%)	20 (55.6%)	15 (48.4%)	0.82
Body mass index (kg/m^2^)	17.65 ± 3.21	17.47 ± 3.05	17.43 ± 3.12	0.71	17.21 ± 3.54	17.30 ± 3.62	17.63 ± 3.49	0.62
SER (D)	− 2.81 ± 1.47	− 2.76 ± 1.56	− 2.66 ± 1.39	0.62	− 2.70 ± 1.79	− 2.65 ± 1.88	− 2.72 ± 1.75	0.58
Intraocular pressure (mmHg)	15.9 ± 3.1	16.9 ± 2.8	17.0 ± 3.0	0.38	15.9 ± 3.1	16.9 ± 2.8	17.0 ± 3.0	0.42
Pupil diameter (mm)	6.12 ± 0.73	6.08 ± 0.59	6.15 ± 0.61	0.42	6.41 ± 0.82	6.19 ± 0.79	6.22 ± 0.89	0.45
Accommodation amplitude (D)	15.92 ± 4.85	15.16 ± 5.06	16.11 ± 5.29	0.67	15.00 ± 8.01	15.34 ± 7.86	15.88 ± 7.23	0.70
Axial length (mm)	24.61 ± 0.69	24.60 ± 0.72	24.54 ± 0.69	0.80	24.58 ± 0.76	24.56 ± 0.79	24.56 ± 0.80	0.76
Anterior chamber depth (mm)	3.69 ± 0.20	3.70 ± 0.20	3.66 ± 0.21	0.92	3.62 ± 0.26	3.74 ± 0.27	3.69 ± 0.31	0.88
Corneal curvature (D)	42.79 ± 1.50	42.81 ± 1.33	42.90 ± 1.09	0.76	42.81 ± 1.56	42.83 ± 1.44	42.94 ± 1.32	0.81
Corneal astigmatism (D)	0.56 ± 0.20	0.56 ± 0.28	0.58 ± 0.29	0.32	0.59 ± 0.22	0.58 ± 0.26	0.59 ± 0.30	0.41
Outdoor activity (hours per day)^※^	2.56 ± 1.41	2.63 ± 1.36	2.60 ± 1.33	0.89	2.63 ± 1.40	2.55 ± 1.29	2.68 ± 1.45	0.76
Near work (hours per day)^#^	14.33 ± 2.02	14.12 ± 1.66	14.54 ± 1.59	0.84	14.14 ± 2.11	14.39 ± 1.90	14.25 ± 2.08	0.79
Heredity				0.99				0.59
+ + (Both parents myopic)	23	24	20		10	11	12	
+ − (One parent myopic)	55	55	45		13	13	14	
− − (Neither parent myopic)	27	27	24		10	12	5	

There were no significant differences observed those who completed and did not complete the 2 years’ study.

*SER* Spherical equivalent refractive error.

^※^Outdoor activity^10,11^ = outdoor exercise + outdoor leisure activity.

^#^Near work^10,11^ = 3 * (homework + reading + playing on cell phone) + 2 * (using computer + playing video game) + 1 * (watching TV).

### Changes in SER and AL over 2 years for 0.02%, 0.01%atropine, and control groups

**Figure 2:**
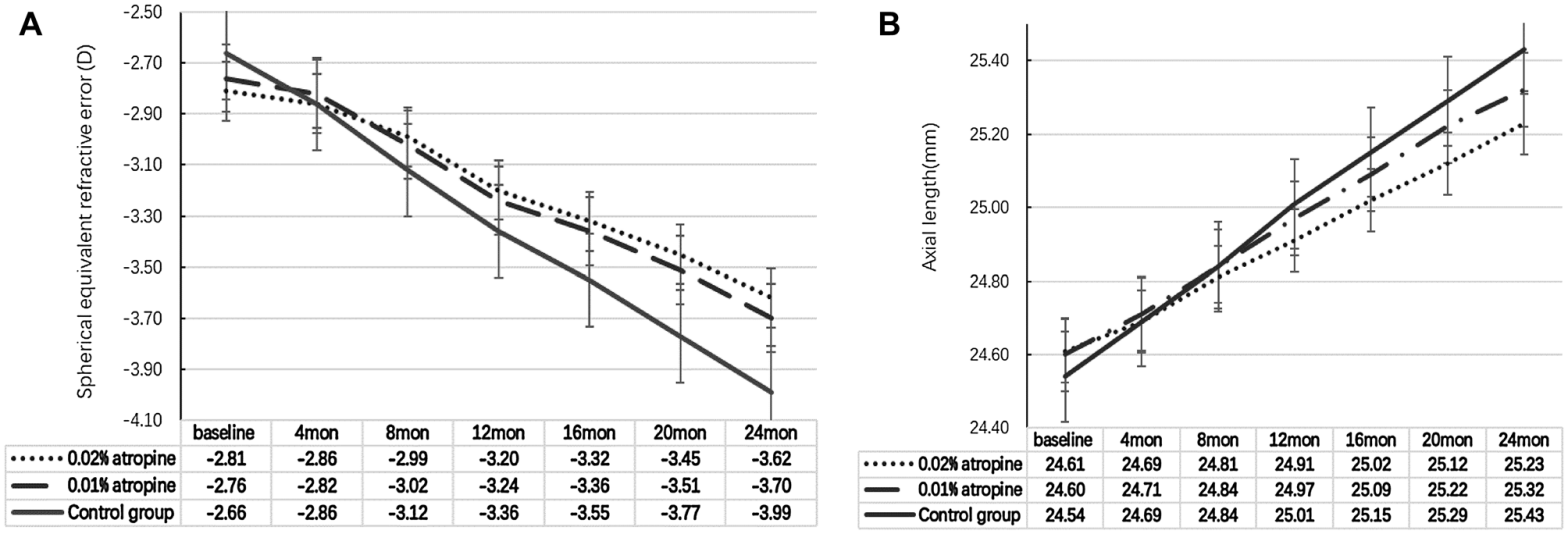
Measurement of spherical equivalent refractive error and axial length over time.

An atropine concentration-dependent response was observed for myopia control after 24 months of treatment. The SER changes were − 0.80 (0.52) D, − 0.93 (0.59) D, and − 1.33 (0.72) D and the AL changes were 0.62 (0.29) mm, 0.72 (0.31) mm, and 0.88 (0.35) mm in the 0.02% and 0.01% atropine and control groups, respectively. There were significant differences between the changes in SER and AL in the three groups (all *P* < 0.05) (Fig. 2A,B and Table 2). In total, 49.5%, 45.2%, and 26.9% of the subjects progressed by less than 1.0 D in the 0.02% and 0.01% atropine and control groups, respectively, whereas 16.2%, 18.8%, and 34.8% subjects progressed by more than 2.0 D in the 0.02% and 0.01% atropine and control groups, respectively.

**Table 2:** Change and change difference of SER and AL in three groups over 2-year.

Variables	Mean (95% CI)									
	0.02% atropine		0.01% atropine		Control group		Change difference between-group			
	Baseline	24 months change	Baseline	24 months change	Baseline	24 months change	0.02% vs. 0.01%atropine	*P* value	0.01% atropine versus control group	*P* value
SER	− 2.81 D (− 2.90 to − 2.72)	− 0.033* (− 0.055 to − 0.011)	− 2.76 D (− 2.81 to – 2.71)	− 0.041* (− 0.066 to − 0.016)	− 2.66 D (− 2.70to − 2.62)	− 0.055* (− 0.091 to − 0.019	0.009 (0.001 to 0.017)	0.03	0.014 (0.003 to 0.025)	0.008
AL	24.61 mm (24.48 to 24.74)	0.025* (0.001 to 0.050)	24.60 mm (24.48 to 24.72)	0.031* (0.003 to 0.059)	24.54 mm (24.41 to 24.67)	0.038 * (0.014 to 0.062)	0.006 (0.001 to 0.011)	0.03	0.008 (0.004 to 0.012)	0.01

CI: confidence interval. SER: spherical equivalent refractive error; AL: axial length.

Change: represents the slope of SER(D/month) and AL (mm/month) over time for three groups.

Change difference: represents the difference in slope of SER and AL over time between each two groups.

A generalized additive mixed model was used to estimate the longitudinal trend from baseline to 24 months. A significant increase was shown in change in SER and AL in three groups from baseline to 24 months.

*Represents: changes were significantly different.

### Comparison of changes of SER and AL in the second year versus the first year

In the 0.02% and 0.01% atropine and control groups, the SER changes were − 0.38 (0.35) D, − 0.47 (0.45) D, and − 0.70 (0.60) D, respectively, during the first year of treatment and − 0.42 (0.32) D, − 0.46 (0.45) D, and − 0.63 (0.59) D, respectively, during the second year of treatment; the AL changes were 0.30 (0.21) mm, 0.37 (0.22) mm, and 0.46 (0.35) mm during the first year of treatment and 0.32 (0.21) mm, 0.35 (0.22) mm, and 0.42 (0.34) mm during the second in the three groups, respectively. The changes in SER during the 1st year were similar to those during the 2nd year in the three groups (all *P* > 0.05), and AL showed a similar trend to that of SER in every year. The correlation between the changes in AL (independent variable) and SER (dependent variable) after the 2-year treatment was − 1.40 (*P* < 0.0001). Multivariate regression analyses after adjusting for baseline SER showed a strong relationship between the changes in AL and SER (β =  − 1.42, 95%CI, − 1.61 to − 1.21, *P* < 0.0001) (Fig. 2A,B, and Table 2).

### Changes in accommodation amplitude and pupil diameter

**Figure 3:**
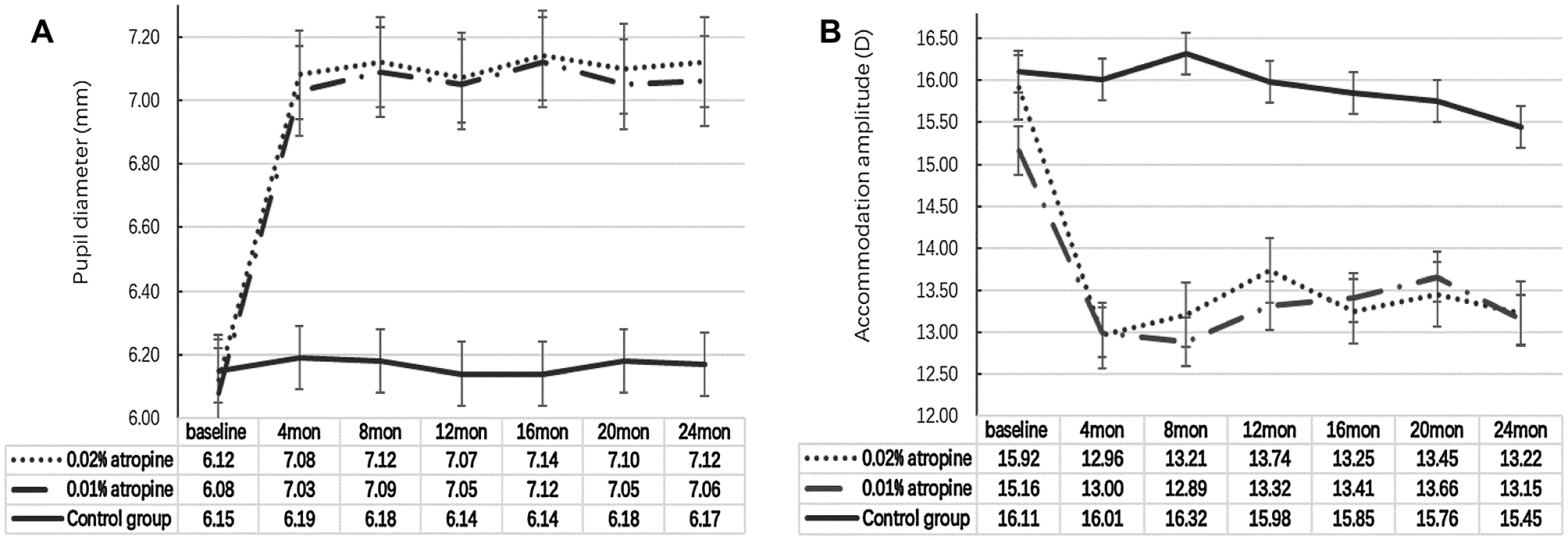
Measurement of pupil diameter and accommodation amplitude over time.

There were no dose-dependent effects of atropine on AMP or PD in the atropine-treated groups. From baseline to 4 months, AMP decreased and PD increased significantly in the two atropine groups (all *P* < 0.001). From 4 to 24 months, AMP and PD remained stable. From baseline to 24 months, the overall changes in AMP (*P* = 0.67) and PD (*P* = 0.51) were not significantly different in the two atropine groups, whereas the AMP (*P* = 0.28) and PD (*P* = 0.19) in the control group remained stable (Fig. 3A,B and Table 3).

**Table 3:** Change and change difference of accommodative amplitude and pupil diameter in three groups over 2-year.

Variables	Mean (95% CI)												
	0.02% atropine			0.01% atropine			Control group	Change difference between 0.02% and 0.01% atropine					
	0–4 months change	4–24 months change	0–24 months change	0–4 months change	4–24 months change	0–24 months change	0–24 months change	0–4 months	*P* value	4–24 months	*P* value	0–24 months	*P* value
Accommodative amplitude (diopters/month)	− 0.71* (− 1.01 to − 0.41)	0.01 (− 0.01 to 0.03)	− 0.11* (− 0.15 to − 0.06)	− 0.57* (− 2.67 to − 1.95)	0.01 (− 0.02 to 0.04)	− 0.09* (− 2.7 to − 0.1)	− 0.03 (− 0.07 to 0.01)	− 0.13 (− 0.32 to 0.06)	0.84	0 (− 0.02 to 0.02)	0.75	− 0.02 (− 0.03 to 0.01)	0.69
Pupil diameter (mm/month)	0.24* (0.17 to 0.31)	0.002 (− 0.001 to 0.005)	0.04* (0.02 to 0.06)	0.24* (0.16 to 0.32)	0.001 (− 0.001 to 0.003)	0.04* (0.03 to 0.05)	0.001 (− 0.001 to 0.003)	0.001 (− 0.002 to 0.004)	0.89	0.001 (− 0.003 to 0.005)	0.91	0 (0 to 0.001)	0.76

CI: confidence interval. Change: represents the slope of accommodative amplitude and pupil diameter over time for three groups.

Change difference: represents the difference in slope of accommodative amplitude (D/month) and pupil diameter (mm/month) over time between each two groups.

A generalized additive mixed model was used to estimate the longitudinal trend.

*Represents: changes were significantly different.

### Adverse events

During 1st year, 32 (23%, 0.02% atropine) and 33 (24%, 0.01% atropine) children were photophobic in bright sunlight, but no other discomfort in normal indoor or daily outdoor light was experienced in either of the atropine groups. In one of the cases, photophobia disappeared during the 2nd year (after 18-month treatment with 0.01% atropine). Photophobia was resolved by wearing sunglasses or sun hats during outdoor activities. No child was allergic to 0.01% or 0.02% atropine or showed any other discomfort associated with atropine during the 2nd year. Three children in the control group were photophobic in bright sunlight for about 2 months in the first year, but had no other discomfort in normal indoor or daily outdoor light.

## Discussion

Our 2-year study showed that once-nightly use of 0.02% atropine had a better effect on myopia control than 0.01% atropine, and its effects on PD and AMP were similar to those of 0.01% atropine for children in mainland China. Treatment with 0.02% or 0.01% atropine controlled myopia progression and AL elongation synchronously and had similar effects on myopia control each year.

Comparisons between the current study and other studies are shown in Table 4. This study showed that 0.02% atropine had a better effect on myopia control than 0.01% atropine over 2 years, which was consistent with the studies in Singapore, Hong Kong China, and Korea^7,10,17^. They all demonstrated that there was a dose-related myopia control response to atropine. The higher the concentration of atropine, the better the myopia progression control. To date, three studies^7,11,12^ reported the efficiency of low-concentration atropine in controlling myopia progression for more than 2 years. The ATOM2 study^7^ in Singapore found that 0.01% atropine was more effective during the second year, as the change in SER was only − 0.06 D during the second year compared with − 0.43D during the first year. During phase 2 of the LAMP study in Hong Kong China^11^, which did not involve a control group during the second year, the efficacy of 0.01% was slightly better during the second than the first year. A multicenter case–control retrospective study in a multiethnic cohort of children using 0.01% atropine in the USA^12^ showed a change in SER of − 0.3 D during the first year and − 0.2 D during the second year, which also showed that the efficacy of 0.01% atropine during the second year was better. They postulated that the better efficacy of 0.01% atropine during the second year was due to cumulative effects over time and suggested that the initial treatment should be continued for at least 2 years^7,11^. However, the efficacies of 0.02% and 0.01% atropine during the second year were similar to those recorded during the first year in the current study. A difference in the increase in the degree of myopia each year after the use of low-dose atropine has not been established yet, and it necessitates further exploration. According to our 2-year results, if the efficiency of 0.01% atropine during the first year is not reasonable, increasing the atropine concentration or combining 0.01% atropine with OK lenses^19–22^ may improve the efficiency of myopia control.

**Table 4:** Control rate of spherical equivalent refractive error (SER) progression and axial length elongation on myopia children using atropine in different studies.

Author, year	Country	Study design	Age (y)	Baseline SER (D)	Follow-up time (M)	Change of SER (D)				Control rate of SER progression (%)			Change of axial length(mm)				Control rate of axial length elongation (%)		
						Control group	Concentration of atropine						Control group	Concentration of atropine					
Current study	Mainland China	RCT	6–14	− 1.25 to − 6.00			0.01%	0.02%		0.01%	0.02%			0.01%	0.02%		0.01%	0.02%	
					0 − 12	− 0.70	− 0.47	− 0.38		32.9	45.7		0.46	0.37	0.30		19.6	34.8	
					12 − 24	− 0.63	− 0.46	− 0.42		27.0	33.3		0.42	0.35	0.32		16.7	23.8	
					0 − 24	− 1.33	− 0.93	− 0.80		30.1	39.8		0.88	0.72	0.62		18.2	29.5	
Chia et al.^7^	Singapore	RCT	6–12	≤ − 2.0			0.01%	0.1%	0.5%	0.01%	0.1%	0.5%		0.01%	0.1%	0.5%	0.01%	0.1%	0.5%
					0–12	− 0.76	− 0.43	− 0.31	− 0.17	43.4	59.2	77.6	0.20	0.24	0.13	0.11	0	35.0	45.0
					12–24	− 0.44	− 0.06	− 0.07	− 0.13	86.4	84.1	70.5	0.18	0.17	0.15	0.16	5.6	16.7	11.1
					0–24	− 1.20	− 0.49	− 0.38	− 0.30	59.2	68.3	75	0.38	0.41	0.28	0.27	0	26.3	29.0
Yam et al.^11^	Hong Kong, China	RCT	4–12	≤ − 1.0			0.01%	0.025%	0.05%	0.01%	0.025%	0.05%		0.01%	0.025%	0.05%	0.01%	0.025%	0.05%
					0–12	− 0.81	− 0.64	− 0.46	− 0.25	21.0	43.2	69.1	0.41	0.35	0.29	0.20	14.6	29.3	51.2
					12–24	–	− 0.48	− 0.39	− 0.30	–	–	–	–	0.25	0.22	0.18	–	–	–
					0–24	–	− 1.12	− 0.85	− 0.55	–	–	–	–	0.59	0.50	0.39	–	–	–
Larkin et al.^12^	American	Retrospective	6–15	− 0.25to − 8.00			0.01%			0.01%									
					0–12	− 0.6	− 0.2			66.7									
					12–24	− 0.6	− 0.1			83.3									
					0–24	− 1.2	− 0.3			75									
Wei et al.^13^	Mainland China	RCT	6–12	− 1.00 to − 6.00			0.01%			0.01%				0.01%			0.01%		
					0–12	− 0.76	− 0.49			35.5			0.41	0.32			22.0		
Clark et al.^14^	American	Retrospective	6–15	− 0.25to − 8.00			0.01			0.01%									
					0–12	− 0.6	− 0.1			83.3									
Moon et al.^17^	Korea	Retrospective	5–14	≥ − 6.0			0.01%	0.025%	0.05%	0.01%	0.025%	0.05%		0.01%	0.025%	0.05%	0.01%	0.025%	0.05%
					0–12	− 1.61	− 0.84	− 0.56	− 0.23	47.8	65.2	85.7	0.55	0.44	0.30	0.23	20.0	45.5	58.2
Joachimsen et al.^16^	German	Retrospective	6–17	SE progression ≥ 0.5 D /year			0.01%			0.01%									
					0–12 M	− 1.05	− 0.40			61.9									
Sacchi et al.^15^	European	Retrospective	5–14	SE progression ≥ 0.5 D /year			0.01%			0.01%									
					0–12 M	− 1.09	− 0.54			50.5									

Few studies have reported the efficacy of low-dose atropine in slowing axial elongation. To date, five studies^7,11,13,17,18^ have reported AL changes after using low-dose atropine (Table 4). In the current 2-year study, the control rates of SER progression by 0.01% and 0.02% atropine were 32.9% and 45.7% during the first year and 27.0% and 33.3% during the second year, respectively. The respective control rates of AL elongation were 19.6% and 34.8% during the first year and 16.7% and 23.8% during the second year. There was a strong association between the changes in AL and SER (β =  − 1.42), suggesting that low-dose atropine controlled myopia progression and AL elongation synchronously. When assessing the efficacy of low-dose atropine in controlling myopia progression in children, both SER and AL must be measured. However, there was a large range of ages and initial SER in the 0.02% and 0.01% atropine and control groups. It has been suggested that lens thinning may have a greater role in emmetropisation (and therefore changes in refractive error) in younger age groups than older^23–25^.The potential influence of the lens and its role may confound the expected relationship between AL and SER in different age groups, which requires further study. Similar results to the current study on the relationship between the changes in AL and SER were reported in Hong Kong China, Korea, and mainland China studies^11,13,17^. However, the ATOM2 study^7^ found that 0.01% atropine only controlled myopia progression but not AL elongation; while the degree of myopia was stable, AL continued to increase between 8 and 24 months after using 0.01% atropine, but this study did not have a proper placebo group to compare AL elongation.

The control of SER progression using atropine varies by race and ethnicity. Studies showed that 0.01% atropine over 12 months controlled SER progression by 50.5–83.3% in White children and 21–59.2% in East Asian Children (Table 4). However, moderate-dose and high-dose atropine may significantly slow myopia progression in Asian children compared with White children^26^. The reasons for the different effects of atropine use by race and ethnicity remain unknown. The remaining question that needs to be clarified in future studies is as follows: “what is the specific mechanism for the race/ethnicity difference in the effects of different concentration atropine?” Further randomized clinical trials should be conducted to confirm the present findings. In the four studies^11,13,17,18^ on East Asian children using 0.01% atropine, the control rates of AL elongation were similar (14.6%, 18.2%, 20%, and 22%), except for the Singapore study^7^ (no effect). Meanwhile, the control rate of AL elongation was less than that of SER progression, whereas AL was not measured in the four studies^12,14–16^ on White children after using 0.01% atropine.

Consistent with other studies^7–17^, there was a higher proportion of major ocular symptoms such as photophobia and near-vision blur during the early stage after using low-dose atropine, then the proportion of ocular symptoms decreased and remained stable. Approximately 17% of the children in both atropine groups had photophobia in bright sunlight but no other discomfort symptoms during the second year. Different proportions of photophobia (0–24%) and near-vision blur (0–5.9%) have been reported by different studies^7–17^. Photophobia may show individual differences, regardless of age, gender, myopic degree, and other parameters^27^. Overall, the major ocular symptoms after using low-dose atropine are mild and tolerable and do not affect the studies and daily activities of children.

The strength of this study is that it used a control group throughout. Although advice from our human ethics committee mandated that subjects were to be offered either atropine or no atropine and double-blinded randomization to be carried out only for the two active arms of the study, the control and test groups had similar demographic and clinical parameters, and the subjects were recruited using identical inclusion criteria, contemporaneously, and from the same population.

In conclusion, our 2-year findings showed that 0.02% atropine had a better effect on myopia control than 0.01% atropine, and 0.01%and 0.02% atropine showed similar effects on PD, AMP, and discomfort symptoms after 24 months of treatment in children in mainland China. The two low concentrations had a similar efficiency for myopia control each year. Individualized corrective methods (such as increasing the atropine concentration from 0.01 to 0.02%, combining lose-dose atropine with OK lenses) should be adopted to improve the myopia control effects in children with unreasonable efficiency after using low-dose atropine for 1 year. Low-dose atropine controlled myopia progression and AL elongation synchronously.

## Methods

Details of the methods and study design have been published elsewhere and are briefly described here^18^. The inclusion criteria were Chinese children aged 6–14 years with myopic SER of − 1.25 to − 6.00 D in both eyes, astigmatism of less than 2.0 D, anisometropia of less than 1.0 D, monocular best-corrected visual acuity of 16/20 or better, and intraocular pressure (IOP) between 10 and 21 mmHg, with no other eye diseases or surgery. Those who had previously used atropine, pirenzepine, or rigid gas-permeable or OK lenses or multifocal contact lens to control myopia progression or were unable to comply with the study schedule were excluded. This study was registered with the Chinese Clinical Trial Registry (registration number: ChiCTR-IPD-16008844, first registration in 14/07/2016) and approved by the Ethics Committee of the First Affiliated Hospital of Zhengzhou University (Approval Number: 2016-35). Under the premise of following the Declaration of Helsinki, all the candidates and guardians provided and signed an informed consent form.

At the randomization visit, eligible subjects were given the option of atropine or no atropine, per human ethics committee of requirements, and the atropine groups were subsequently assigned in a double-blinded and randomized manner either to 0.01% or 0.02%. All subjects were prescribed best-corrected spectacles for constant wear during the day. The children in the atropine treatment group received 0.01% or 0.02% atropine eye drops in both eyes once every night. The 0.01% and 0.02% atropine eye drops (pH = 5.4–5.6, 3 mL sealed bottle, 15–25 °C room temperature storage, discarded eye drops after opening the bottle for 1 month) were prepared by diluting 1% atropine eye drops (Eye and ENT Hospital Affiliated to Fudan University) with 0.9% normal saline under sterile conditions and subsequently, adding a preservative (0.3 mg/mL ethylparaben). The atropine eye drops were degraded less (about 1.8%) after opening the eye drops bottle for 1 month and its properties were relatively stable. The shelf life was over 6 months. Neither the examiner nor the subject knew the concentration of the eye drops. All eye drops were kept and distributed by the same doctor.

Standardized ophthalmological examination for all subjects was performed at baseline, at the 1-month monitoring visit, and then every 4 months until 2 years after treatment. Examinations in the second year were the same as those in the first year as described previously. All examinations were performed by the same physician in the morning. Axial length (AL), corneal power, and anterior chamber depth (ACD) were evaluated using a non-contact partial coherence interferometer (IOLMaster; Carl Zeiss Meditec AG, Germany). On each occasion, five successive measurements were taken and their mean was used for analysis. The pupil diameter was measured with an autorefractor (NIDEK, AR-1, Japan) under bright light indoors. The light in the examination room was constant with the illumination of 300 to 310 lx (TES-1332A illumination photometer). The children had to adapt to the ambient light in the examination room for 10 min before the measurement. Three consecutive measurements were taken, and the average value was recorded. Accommodation amplitude (AMP) was measured monocularly by the push-up technique. The children wore their fully corrected spectacle prescription and focused on the previous line of best-corrected visual acuity with the right eye while the left one was occluded. The children were instructed to focus on a letter as the chart was moved closer. They were told to keep the letter as clear as possible until it could no longer be held in clear focus. The inverse of the final distance in meter was recorded as the child’s AMP. AMP was recorded three times and the average taken. Discomfort symptoms in the experimental groups were assessed using a paper questionnaire^18,24^ during each follow-up visit. Cycloplegic autorefraction was performed using four drops of compound tropicamide eye drops^28,29^ (0.5% tropicamide and 0.5% neo- synephrine) (Santen, Japan) administered to both eyes at an interval of 10 min. Ten minutes after the last drop, cycloplegic autorefraction was measured three times by an autorefractor (Topcon RM 8000A, CA). Three readings, all within a difference of 0.25 D, were averaged for analysis. SER was calculated as the sphere plus half of the cylindrical power.

Continuous baseline variables were expressed as mean (SD) and evaluated by analysis of variance. Categorical variables, such as sex and parental myopia status, were expressed as percentages (%) and evaluated using the Chi-squared test. A generalized additive mixed model was used to estimate the longitudinal trend with time (baseline, 4, 8, 12, 16, 20 and 24 months) for dependent variables (SER, AL, PD, and AMP) and the differences in the rate of change between the three groups. The change represents the slope for each treatment group of dependent variables over time, and the change difference represents the difference in the slopes of the dependent variables over time of the groups. A *P* value < 0.05 was considered statistically significant. All statistical analyses were performed using Empower (WWW. EMPOWERSTATA COM; X & Y Solutions, Boston, MA) and R.
